# Book Review

**Published:** 1923-10

**Authors:** 


### Reviews of Books.

**An Index of Prognosis and End-Results of Treatment.** By various Writers. Edited by A. Rendle Short, M.D., B.S., B.Sc. Lond., F.R.C.S. Eng. Third Edition, revised and enlarged. Bristol: John Wright & Sons Ltd. 1922.—We heartily welcome the appearance of this new edition of a work so well known and widely appreciated, which has already proved itself an invaluable guide both as to prognosis and as to the value of different forms of treatment. In the seven years since the first edition was published much has been learnt, especially from the results of the Great War. The work has now been carefully revised and in many parts rewritten, and a new section on Ophthalmology added. Laborious as the compilation of the original work was, the new statistics available and fresh means of prognosis have added immensely to the duties of the editor in the present edition.

**Hæmatology in General Practice.** By A. Knyvett Gordon, M.B., B.Ch., B.A. Pp. vii., 100. London: Baillière, Tindall & Cox. 1923. Price 5s.—The author of this little work has been most successful in the accomplishment of the task he has set out to achieve, viz. to enable the practitioner to utilise the light which a rough examination of a blood film may throw on the diagnosis and prognosis of certain diseases. The chapters on the biology of blood cells, the scope of Hæmatology, the normal blood picture, and the genealogy and pathology of the red and white cells, are concise, definite, and well written, and cannot fail to be of great help both to the student in his ward work and the general practitioner in his every-day routine. The book is well printed, the illustrations are good, and the subject-matter is dealt with in a lucid and intelligible way, which cannot fail to commend it to those for whom it is primarily intended.

**The Clinical Examination of the Nervous System.** By G. H. MONRAD-KROHN, M.D. (Christiania), M.R.C.P. (Lond.) Second Edition. Pp. xvi., 148. London: H. K. Lewis & Co. Ltd. 1923. Price 6s. net.—Professor Monrad-Krohn is to be congratulated on his signal success in writing a book in a foreign tongue (for it is not a translation, but an English book written by a Norwegian), which needs a new edition within two or three years of its initial publication. Although he has compressed a surprising bulk of information into a small compass, the book is easy to read, and full of practical suggestions and hints. How many British professors of medicine are there who could accomplish a corresponding feat ?

**Hernia and its Radical Cure.** By J. HUTCHINSON, F.R.C.S. Eng. Pp. xiii., 264. London: Oxford Medical Publications. 1923. Price 12s. 6d. net.—While this book deals with hernia chiefly from its practical operative point of view, the author expresses very definite opinions on the present-day theories of the origin of this disability, and brings out numerous points in treatment and prognosis not always within the knowledge of those whose experience is not large. He condemns the routine practice of one operation for one type of hernia as being, in some cases, unsatisfactory, and likely to bring disrepute upon methods which, suitably applied, give excellent results, with, at the utmost, 10 per cent. only of failure to cure radically ; while some modern innovations in radical cure which savour more of advertisement than advancement the author deals with in no uncertain voice. The rarer forms of hernia are all adequately dealt with, while to strangulation, its diagnosis and treatment is assigned a considerable portion of the book, giving many valuable details of the difficulties which the surgeon of wide experience has met and overcome in this condition. Any attempt to describe the varieties and application of trusses has been wisely avoided. This book offers a useful survey of the radical cure of hernia, pointing out its difficulties and the need for careful selection of methods and attention to details.

**Surgical "Don'ts" (and "Do's").** By C. HAMILTON WHITEFORD, M.R.C.S. Pp. 46. London: Harrison & Sons Ltd. 1923. The author deserves the thanks of surgeons for broadcasting rules of professional ethics and etiquette otherwise only learnt by years of often painful experience. He warns us that a surgeon and his scalpel should not be inseparable. The work resounds with a fine frank moral note calculated to inspire a lofty ideal in the profession, and to increase the admiration and confidence of the laity whom it serves.

**Problems in Tuberculosis.** By Sir James Kingston Fowler, K.C.V.O., M.A., M.D. Pp. ix., 64. London: Oxford Medical Publications. 1923. Price 5s. net.—This excellent little book, from the pen of one of the greatest authorities on tuberculosis, is full of interest from cover to cover. Kingston Fowler is a keen opponent of tuberculin, and and has some cogent remarks to make *à propos* of tuberculosis dispensaries. "If some tuberculosis dispensaries had been more concerned with contacts and less with tuberculin things might have been different." On the other hand, he has much to say in praise of Otto Walther and Marcus Paterson in relation to treatment by auto-inoculation through the medium of graduated labour. He has some sound remarks to make on the subject of duration of treatment in curable cases. There is much time and money spent to no purpose in giving short spells of treatment to cases that can never materially benefit, and the treatment of hopeful cases is of necessity curtailed in consequence. He puts the problem in a nutshell: "For income 5 per cent. on £100 is better than no dividend on £200." The author has some damaging criticisms to make on mistakes in diagnosis and notification which are very suggestive. The chapters on organisation of the tuberculosis settlement and advice to tuberculous patients with regard to their future are illuminating, full of interest and very sound. A not inconsiderable merit of the book is its brevity; it can be read in a couple of hours, and there is no time for interest to flag.

**Hæmorrhoids, their Etiology, Prophylaxis and Treatment by Means of Injections.** By Arthur S. Morley, F.R.C.S. Eng. Pp. ix., 114. London: Oxford Medical Publications. 1923. Price 6s.—Mr. Morley is convinced that the usual operations performed for the cure of hæmorrhoids are rarely necessary, and that treatment by interstitial injections should largely replace those operations. The injection treatment saves the patient from lying up, from having an anæsthetic, from considerable pain and from much inconvenience and expense. The author gives a full account of the technique of the procedure and of its indications, contra-indications and results. It is a curious thing, if others can obtain as good results by this treatment as the author, that the method has not gained greater popularity. Is it an example of unreasonable conservatism? It is obvious that the method is more troublesome to the surgeon than one operation, for he has to make the injections four to six times in each case. We hope that fact has not been allowed to influence opinion unduly. We think rather that the conservatism is based on the fact that the ordinary operative treatment of hæmorrhoids yields very satisfactory results, results comparing favourably with those obtained from any surgical operation in whatever field. Hence but little desire is felt to try newer methods, the preference being to bear the ills of present methods rather than fly to others that may attend a newer and at first sight less radical method. After reading Mr. Morley's book and other articles on the subject, such as Mr. Dudley Wright's pamphlet of some ten or twelve years ago and Sir James Goodhart's article in *The Practitioner* of 1914, we cannot but feel that the injection treatment of hæmorrhoids has a great future, and we highly commend the clear account of it here given.

**An Index to General Practice.** By A. CAMPBELL STARK, M.B., B.S. Pp. x., 181. London: Baillière, Tindall & Cox. 1923. Price 5s. net.—This book is not an encyclopædia but a medical experience unrolled. "Antiseptics," "Books," "Clinical Observation," Dr. Stark deals with work as single hands encounter it. Here is the craftsman, ingenious in device, whether to keep records or to specialise; and the discriminating reader who, brief himself, can tell the pithiest author on each topic. Dr. Stark's *forte* is Materia Medica, and his formulæ will be scrutinised for alopecia, common colds and puerperal sepsis. But he has no weak bias for drugs and recognises that treatment lags behind diagnosis. We miss the old latinity from his prescriptions and could spare a dead fly of acerbity in his wisdom. The author disputes the right of the schools to engross education and dictate opinion. To general practice, too, it belongs—and this gives consistency and aim to his book —to shape the novice, hold the field of endeavour and make gains for knowledge.

**A System of Surgery.** Edited by C. C. CHOYCE, C.M.G., C.B.E., M.D., B.Sc., F.R.C.S. Pathological Editor, J. MARTIN BEATTIE, M.A., M.D., C.M. In three volumes. Second Edition. London: Cassell & Co. Ltd. 1923. £6 nett.—It has long been an open secret that a second edition of this standard text-book of systematic surgery was in preparation; it may be said at once that the inevitable delay in its appearance is amply compensated for in the completeness of the revision. Nothing seems to have been overlooked in the attempt to provide a complete and up-to-date exposition of British surgery. The majority of the contributors are the same as were responsible for the first edition, and the names of the newcomers are a sufficient guarantee of the maintenance of the high standard then reached. Where all the sections are so complete it would be invidious to select one or more for special mention. It may be stated with confidence that the reader in search of adequate information on any subject within the compass of the work is not likely to be disappointed. To give an example, Nitch's contribution on gangrene contains an account of Leriche's work on the prophylaxis of senile and other forms of gangrene by stripping the sympathetic coat from the wall of the artery. Many similar examples of this completeness might be quoted. A noteworthy feature of great value is the way in which a fair summary of present-day opinion on subjects of a controversial nature is presented to the reader, no attempt being made to stress unduly the writer's personal views. No better example of this can be given than Hey Groves's section on fractures, to which the Bristol reviewer turns with natural curiosity. This difficult subject is handled in an admirable way, and no attempt is made to exaggerate unduly the merits of the newer methods of treating fractures at the expense of those which, if under a cloud at the moment, have served us well in the past. At the same time, the reader in search of the latest operative or other treatment will find here all he wants. In congratulating Editor and contributors on the success of their efforts, we must not omit to pay tribute to the publishers for their share. The printing and general get-up of the volumes are beyond praise, and the illustrations, both half-tone and coloured, are beautifully produced. In brief, the three handsome volumes thoroughly deserve to keep the place they have won as a standard text-book of systematic surgery as British surgeons see it.

**Practical Anæsthetics.** By CHARLES F. HADFIELD, M.B.E., M.A., M.D. Pp. ix., 244. London: Baillière, Tindall & Cox. 1923. Price 7s. 6d. net.—Dr. Hadfield's book is an extremely useful addition to that small library of anæsthetic text books for students. In these days of packed curricula the size of a text book has an important bearing on its use and popularity, and this small manual of 240 odd pages has succeeded in getting all the essentials of practical anæsthesia within this modest limit. We therefore commend this book both for its price and contents to all students and practitioners.

**Orthopedic Surgery.** By Sir ROBERT JONES, K.B.E., C.B., F.R.C.S., and ROBERT W. LOVETT, M.D., F.R.C.S. Pp. xv., 699. London: Oxford Medical Publications, 1923. Price 42s. net.—A book written by the leading surgeons in this speciality of this country and of the United States is certainly one which will command immediate interest and become an authoritative book of reference. The authors apologise for the use of the term orthopedic, and promise to use it as little as possible, but they had not the courage to abandon it in favour of the simpler term "Surgery of the Bones and Joints." The work is simple and practical, and as such it will be valued as representing the views of the greatest experts on matters of treatment. There is no attempt to make the work inclusive of all that has been written on the subject. It is a plain statement of the authors' own views with occasional reference to some idea or method which has their approval, even though they themselves have not practised it. The work opens with a general discussion on Joints, their function and disabilities, and this leads on to a series of chapters on traumatic tuberculosis and other diseases of the special joints. Coxa plana is included in the section on traumatic affections of the hip and treatment by rest and occasional fixation is advised for it. In injuries of the knee joint the special operations devised by Edwards and Hey Groves for the repair of the lateral and crucial ligaments respectively are fully described and figured. The internal derangements of the knee are dealt with on the usually accepted principles, and it is advised that a torn cartilage should be removed through a small lateral incision, taking away the whole meniscus. We think that more space might have been given to the method of dealing with cases of doubtful nature, or of how to proceed when the lateral incision discloses a normal cartilage. The patella splitting method is not mentioned, although it is being used more and more as an exploratory route in this country, and that chiefly on the recommendation of one of the authors of this book. The treatment of stiff joints by manipulation and splinting is dealt with at some length, whilst the section on arthroplasty is very short and non-committal. Tuberculosis of the joints and spine occupies about 120 profusely illustrated pages. This section is practical and authoritative, and has the distinctive advantage of showing two distinct opinions about the details of treatment, Jones advocating the Thomas appliances with fixed traction and Lovett the more mobile system of weight and pulley. The brace, frame and plaster methods of spinal immobilisation are fully described, and the osteoplastic operations of Hibbs and Albee are epitomised and definitely recommended for adoption in the majority of adult cases. In the treatment of deformities of the leg bones we are rather surprised to find that the osteoclast is still recommended, and two varieties of this machine are figured. The new growths of bone are described in seven pages, and this short summary is intended for assistance in connection with other disabilities of the joints. Recent fractures are not described, but there is a short chapter on mal-united fractures, in which the use of Thomas's wrench, the abduction frame and the malleable iron splints is introduced. In the treatment of spastic paralysis the authors give the first place to muscle re-education, preceded if necessary by tenotomies or tendon lengthening and transplantation. In describing the treatment of infantile paralysis the prevention of deformities by early rest and splinting and the treatment of late deformities by muscle transplantation are given very fully and are illustrated well. The hazardous and difficult operation of lengthening a shortened femur is given much more prominence than it deserves. Congenital dislocation of the hip is fully described in regard to its anatomy and manipulative treatment. Open operation is mentioned with so little detail as to leave the impression that it is hardly worth while considering, and osteotomy of the femoral neck in adults is recommended as a very exceptional measure. The last section of the work deals with club foot, static deformities of the feet, scoliosis and the principles of apparatus. The wrench, retentive apparatus and muscle education are the dominant notes in all these chapters. Finally, we should like to summarize our impressions of the book as a whole. It would be impertinent to say that the considered opinions of two master minds are anything but good—it is only a question whether it is as good as we might have expected—if it falls short of the ideals inspired by personal contact with the authors we suppose that this can hardly be a fault charged against the book. A printed sermon never conveys the message given by the living man.

**Urgent Surgery.** By Felix Lejars. Third English Edition. Translated by William S. Dickie and Ernest Ward. Pp. xv., 808. Bristol: J. Wright & Son Ltd. 1923. Price 63s.—The third English edition of this valuable book represents the eighth French edition, and instead of being in two volumes as previously, we have the whole work here under one cover. It is a book not only to read, but also to possess. Its great virtue for the surgeon lies in the excellent and full accounts it gives of the means of dealing with the rarer emergencies, some of which are scarcely at all described elsewhere. Moreover, it is very fully illustrated, and the fact that we are seeing surgery through French eyes gives a touch of freshness and occasionally of novelty. The changes in this edition are not very obtrusive. They are principally concerned with the newer methods of treatment which have been taken over from military into civilian surgery, as for extensive wounds of soft parts, hemorrhage, ligature of vessels, and fractures.

**The "Nauheim" Treatment of Diseases of the Heart and Vessels in England.** By Leslie Thorne Thorne, M.D. Sixth Edition. Pp. vii., 232. London: Baillière, Tindall & Cox. 1923. Price 7s. 6d. net.—This book is written to demonstrate the use of these baths, and to show that they may just as well be given in nursing homes or in the patient's own house as at Nauheim. We learn on page 14 that in cases of lowered blood pressure the arterial tension may be raised by immersion in a "Nauheim" bath, whereas in cases of increased arterial tension the blood pressure is lowered. Wonderful results are given in decreasing cardiac dilatation, and the author quotes examples where the cardiac dullness at the nipple level has been reduced from 9 to 4½ inches. We note that the author relies on percussion for outlining the cardiac dullness, and states that the results of percussion agree with those of X-ray findings, whereas the modern cardiologist owns that percussion is very inaccurate. On page 5 it is stated that auricular or ventricular premature contractions show the existence of impaired conductivity; but of this no proof is offered by the author. On page 65 Case II. is shown to have an increased a–c interval, which is stated to have become normal after treatment, but careful measurements of tracings 12 and 13 show that the same lengthening of the a–c interval existed after treatment as before. Figures 54, 57, and 64 are stated to be examples of auricular premature contractions, but in reality they are ventricular in origin. The author states that there is no fear of sudden death in angina pectoris unless there be dilatation, yet we have no doubt that many cases of angina die without this condition. The author is evidently a great enthusiast for the use of these baths.

